# Probing the Relationship Between Home Numeracy and Children's Mathematical Skills: A Systematic Review

**DOI:** 10.3389/fpsyg.2020.02074

**Published:** 2020-09-18

**Authors:** Belde Mutaf-Yıldız, Delphine Sasanguie, Bert De Smedt, Bert Reynvoet

**Affiliations:** ^1^Brain and Cognition, Faculty of Psychology and Educational Sciences, KU Leuven, Leuven, Belgium; ^2^Research Centre for Learning in Diversity, HOGENT, Ghent, Belgium; ^3^Parenting and Special Education, Faculty of Psychology and Educational Sciences, KU Leuven, Leuven, Belgium; ^4^Faculty of Psychology and Educational Sciences, KU Leuven Kulak, Kortrijk, Belgium

**Keywords:** home numeracy, mathematical skills, children, systematic review, *p*-curve analysis

## Abstract

The concept of home numeracy has been defined as parent–child interactions with numerical content. This concept started to receive increasing attention since the last decade. Most of the studies indicated that the more parents and their children engage in numerical experiences, the better children perform in mathematical tasks. However, there are also contrasting results indicating that home numeracy does not play a role or that there is a negative association between the parent–child interactions and children's mathematics performance. To shed light on these discrepancies, a systematic review searching for available articles examining the relationship between home numeracy and mathematical skills was conducted. Thirty-seven articles were retained and a *p*-curve analysis showed a true positive association between home numeracy and children's mathematical skills. A more qualitative investigation of the articles revealed five common findings: (1) Advanced home numeracy interactions but not basic ones are associated with children's mathematical skills. (2) Most participants in the studies were mothers, however, when both parents participated and were compared, only mothers' reports of formal home numeracy activities (i.e., explicit numeracy teaching) were linked to children's mathematical skill. (3) Formal home numeracy activities have been investigated more commonly than informal home numeracy activities (i.e., implicit numeracy teaching). (4) The number of studies that have used questionnaires to assess home numeracy is larger compared with the ones that have used observations. (5) The majority of the studies measured children's mathematical skills with comprehensive tests that index mathematical ability with one composite score rather than with specific numerical tasks. These five common findings might explain the contradictory results regarding the relationship between home numeracy and mathematical skills. Therefore, more research is necessary to draw quantitative conclusions about these five points.

## Introduction

According to Vygotsky's sociocultural theory (Vygotsky, 1978), culture, community, and social interactions play a fundamental role in children's learning and development. This theoretical framework indicates that people, such as teachers, peers, siblings, and parents, who are present in the environment of children have the power to (educationally) stimulate the children by social interactions, and this process is influenced by environmental factors such as culture and socio-economic status. Especially, the past few years, increasing attention has been paid to understand the learning opportunities brought by parents that affect children's mathematical skills. More specifically, the concept of “*home numeracy*” has been introduced to describe the various ways in which parents can influence their children's mathematical skills (Blevins-Knabe and Austin, 2016). Most commonly, home numeracy has been operationalized as parent–child interactions related with numerical “*activities*,” such as the frequency of engaging in certain numerical activities or the frequency of using numerical words during certain activities. To a lesser extent, as a part of a home numeracy measure, some researchers also included other indexes, such as parents' academic *expectations* from their children (e.g., Kleemans et al., 2012) and their own *attitudes* toward mathematics (e.g., Skwarchuk et al., 2014). However, in this review we use the term “home numeracy” to specifically refer to the parent–child interactions related with numerical “*activities*” and report findings related to activities only, and not on expectations or attitudes.

Many studies have demonstrated that home numeracy positively correlates with how well-children perform in mathematical tasks (e.g., Blevins-Knabe and Musun-Miller, 1996; LeFevre et al., 2009; Kleemans et al., 2012). On the other hand, some studies reported that home numeracy negatively correlates with children's mathematical abilities (e.g., Blevins-Knabe et al., 2000; Ciping et al., 2015) or has no relation with these at all (e.g., Zhou et al., 2006; Missall et al., 2015). Bearing in mind these contradictory findings, revealing a clear picture of the relationship between home numeracy and mathematical skills is especially important because of the educational impact home numeracy has been suggested to have. First, home numeracy is assumed to explain the variation in kindergartners' early mathematical performance (e.g., LeFevre et al., 2009), which in turn has been shown to affect later mathematical achievement (e.g., Duncan et al., 2007; Gilmore et al., 2010; Sasanguie et al., 2012, 2013). Yet, literature on home numeracy is still in its infancy and lacks a systematic analysis of the available data. Because in the literature there is an abundance of definitions of both home numeracy and mathematical skills, we will first present a clear description of the concepts of home numeracy and mathematical skills.

### Home Numeracy

Different types of home numeracy activities were introduced by LeFevre et al. (2009) based on an analogy with their previous work studying the relation between home *literacy* activities and children's early *literacy* skills (e.g., Sénéchal and LeFevre, 2002). LeFevre et al. (2009) developed a comprehensive questionnaire to assess home *numeracy* via parental self-reports on the frequency of various activities they performed with their child within in a given time frame, e.g., during the last month. Meanwhile, this questionnaire has been used widely. Based on a principal components analysis, the authors suggested that home numeracy activities can be divided into two broad categories, i.e., formal and informal activities. *Formal activities* are defined as parents' intentional teaching efforts, such as counting objects, practicing simple sums, or reading number story books. *Informal activities*, on the other hand, consist of parents' unintended teaching that takes place during activities such as playing board or card games, using calendars and reading clocks. LeFevre et al. (2009) reported that both types of activities positively correlate with children's performance in mathematics.

As already mentioned, the origin of the interest in home numeracy goes back to Vygotsky's (1978) sociocultural theory stating that social interactions play a fundamental role in the development of children's cognitive skills. Hereby, Vygotsky emphasized that children's learning is most efficient when knowledgeable others, such as teachers or parents, can identify the level of children's achievement and build their interactions on top of that level, an idea better known as the “Zone of Proximal Development (ZPD).” In accordance with the idea of ZPD, it has been suggested that home numeracy activities can be further divided into two categories based on difficulty level: basic and advanced activities (Skwarchuk, 2009; Skwarchuk et al., 2014). The distinction between basic and advanced activities of course depends on children's age and performance level. For instance, *basic activities* describe easier number practices, such as counting or recognizing written numbers, whereas *advanced activities* rather refer to more difficult number practices, such as teaching calculations, for ~5-year-olds. Therefore, it can be expected that practicing basic activities that children can already do by themselves does not result in improvement; however, practicing advanced activities that are just beyond children's achievement level provides opportunities for improvement.

Another common method to assess home numeracy is through observation (e.g., Levine et al., 2010). Typically, in an observation study, parent–child dyads are observed while they engage in either daily routine activities at home, such as making dinner (i.e., unstructured observations), or during pre-specified activities, such as book reading or playing with Lego blocks, that are preset by researchers at home or in laboratories (i.e., semi-structured observations). In a next step, the recordings are transcribed to reveal the frequency of the numeracy talk. In other words, observation studies do not investigate how often parents and their children engage in numerical activities; instead they focus on how often parents and their children utter numerical words during certain activities. Furthermore, the numeracy talk can be classified based on its content. For instance, Gunderson and Levine (2011) categorized different types of numeracy talk, such as counting objects and naming cardinal values of objects. Classification of numeracy talk also allows for a distinction similar to basic and advanced activities. For instance, *basic numeracy talk* (e.g., about numbers smaller than 4) and *advanced numeracy talk* (e.g., about numbers larger than 4) can be distinguished relative to children's age (e.g., Gunderson and Levine, 2011).

Although numeracy-related interactions have been proposed as a unique predictor of children's mathematical skills, Anders et al. (2012) observed that not only home numeracy but also home literacy–related interactions were associated with children's mathematical skills. Therefore, some researchers argued that home literacy and numeracy environments are not completely independent from one another, but rather form a more global construct, i.e., the *Home Learning Environment* (HLE; Melhuish et al., 2008; Dearing et al., 2012; Baker, 2015; Niklas and Schneider, 2017). The HLE is most commonly assessed via questionnaires asking about a wider range of activities, both numerical and non-numerical (e.g., frequency of going to library, painting, drawing, or playing dice games), and family possessions (e.g., number of books or access to educational software at home).

Another factor that affects both parents' engagement in home numeracy activities (e.g., Starkey et al., 1999) and children's mathematical skills (e.g., Siegler and Ramani, 2008) is socio-economic status (SES), mostly operationalized as parents' education level and household income (e.g., Jordan et al., 2006; Dubow et al., 2009). It has been suggested that home numeracy activities are related with SES (Niklas and Schneider, 2017; see for a review, Elliott and Bachman, 2018). A recent meta-analysis has documented that the positive associations of home numeracy activities were larger in high-SES families compared with low-SES families (Dunst et al., 2017). However, the direction of the relation is not very clear, as also negative associations between parents' education level and their engagement in home numeracy activities have been reported (e.g., LeFevre et al., 2010a; Niklas and Schneider, 2014). Therefore, we will include SES as a sample characteristic in this review.

### Mathematical Skills

In the present review, “mathematical skills” refer to a wide range of skills (e.g., non-symbolic and symbolic number discrimination, counting, and arithmetic) that studies have used to correlate with home numeracy. Theoretically, mathematical skills can be divided into two categories; *informal* and *formal* (Baroody and Ginsburg, 1990). *Informal skills* are basic skills that are learned through everyday activities, such as non-symbolic number processing and counting skills (e.g., Purpura et al., 2013). Non-symbolic number processing is suggested to rely on an innate ability to approximately represent, understand and manipulate non-symbolic (e.g., dot arrays) magnitudes (Dehaene, 2001). This representation is typically measured with a non-symbolic comparison task, i.e., a task in which participants have to indicate the larger of two dot arrays (e.g., Piazza et al., 2004) or with a non-symbolic number line estimation task, i.e., a task in which participants have to place a dot array on an empty line going from, e.g., 0–10 dots (e.g., Sasanguie et al., 2012). Another informal basic skill is counting. It is assumed that verbal rote counting is first practiced as a routine by mimicking others such as parents and teachers, or by singing counting songs (Wynn, 1990) at an age of around 2. However, understanding that each number in the counting list represents one and only one entity in the sequence, i.e., the so-called “one-to-one correspondence,” and that the last number in the counting list represents the total number of entities in a set, i.e., the “cardinality principle,” are skills that are only acquired from around the age of three and a half (Gelman and Meck, 1983; Wynn, 1990). All these different subskills have been commonly measured with different tasks, such as verbal counting, i.e., counting as high as possible (e.g., Geary et al., 2018) and give-a-number tasks, i.e., choosing a given number of entity from a set (Wynn, 1990).

*Formal skills* are more advanced and require knowledge of symbolic numbers, such as Arabic digits and written number words, which are learned through cultural education and/or direct instructions. Examples of formal skills are for instance symbolic number processing and arithmetic (Baroody and Ginsburg, 1990). Symbolic number processing is the ability to understand and manipulate digits and number words (Dehaene, 2011). Often, this skill is measured with symbolic versions of the comparison task (e.g., Bugden and Ansari, 2011) and number line estimation task (e.g., Siegler and Booth, 2004) that were explained earlier. Another formal, mathematical skill—which is also commonly addressed in home numeracy studies—is arithmetic. Measures of arithmetic skills can tap into accuracy, i.e., solving as many questions correctly, and/or fluency, i.e., solving questions correctly as fast as possible, or making calculations. All these formal and informal skills can be examined with separate specific tasks, but they also can be part of a mathematical test of which performance is then averaged into a composite score. For instance, the Test of Early Mathematics Ability (TEMA, Ginsburg and Baroody, 1990) consists of two subtests: one for formal and one for informal mathematical abilities. The informal subtest measures concepts of relative magnitude and counting skills, whereas the formal subtest measures skills, such as calculation, reading and writing numerals, knowledge of number facts, calculation algorithms, and base 10 concepts. The majority of studies have investigated the role of home numeracy evaluating children's performance based on a composite score of either each subtest separately or both subtests combined.

### The Present Study

Home numeracy on the one hand and mathematical skills on the other hand have been operationalized and measured in various ways in different studies. Therefore, in the current systematic literature review, we aimed to systematically investigate the relationship between home numeracy and children's mathematical skills, by taking the diversities in how both constructs were measured into consideration. Furthermore, to test whether this relationship has a true effect or the findings of the reviewed studies were result of a publication bias, we conducted a *p*-curve analysis (Simonsohn et al., 2013, 2014).

## Method

**Figure 1:**
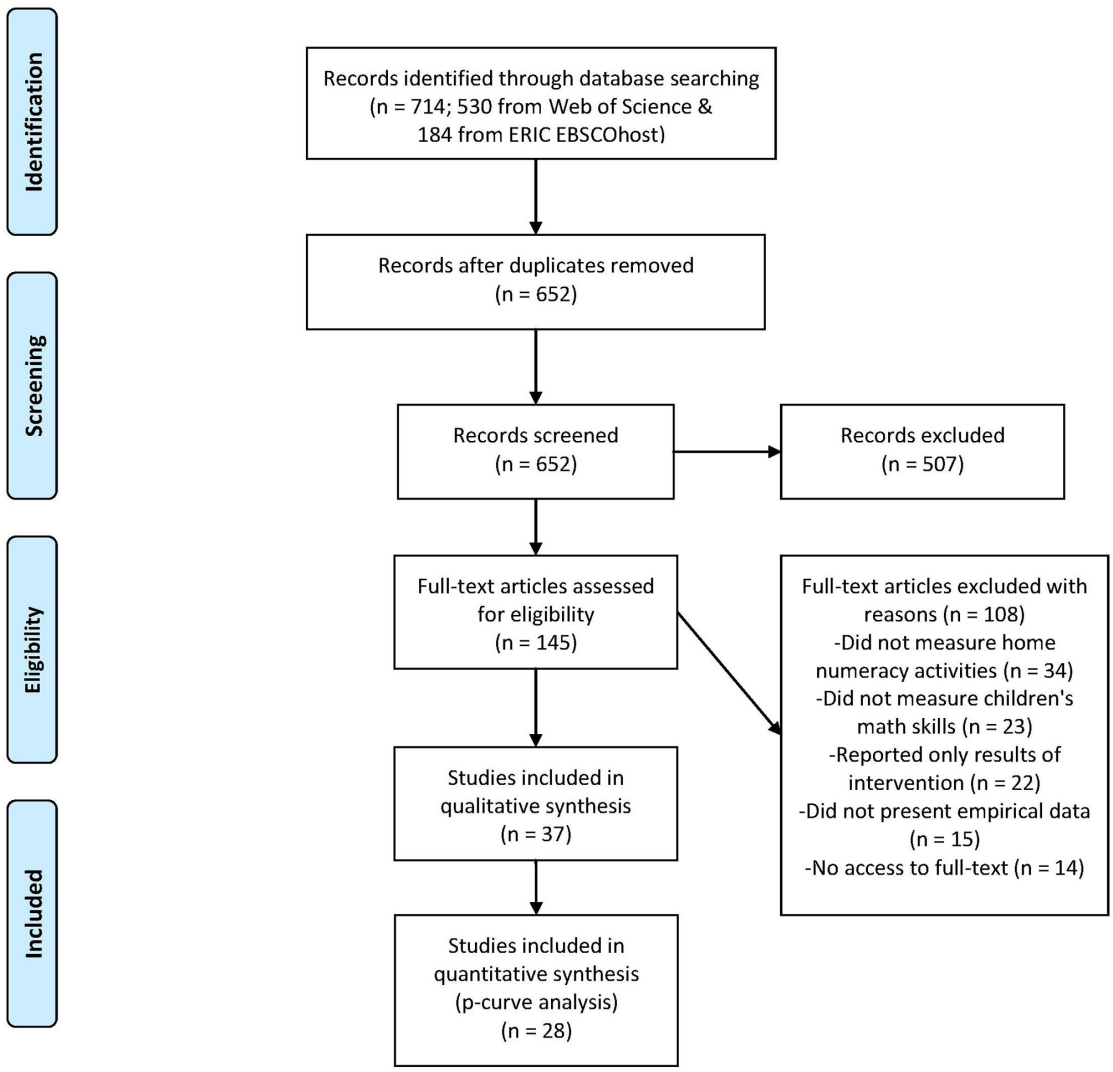
PRISMA flowchart illustrating the article selection procedure through the systematic review process.

A systematic review study (Grant and Booth, 2009) was conducted to obtain a comprehensive overview of the studies that investigated the relationship between home numeracy and children's mathematical skills. Articles were selected in four steps: First, the two most important databases in the field of psychological and educational research were scanned to reach all relevant articles as possible. Second, the abstracts of the articles were screened by three independent raters (i.e., the first, second and fourth author of this article) taking into account the inclusion and exclusion criteria that are described later. Third, the selected articles were read in full and screened taking into account the additional exclusion criteria described below. Lastly, the final subset of articles was analyzed in detail. An overview of the selection process is presented in Figure 1.

### Systematic Search

The online databases ERIC (EBSCOhost) and Web of Science (SSCI) were scanned to reach potentially relevant articles. Our search strategy was limited to peer-reviewed journal articles written in English. We searched for all articles that were published up to November 2019. The search string was as follows: (child^*^ OR kindergart^*^ OR preschool^*^) AND (numer^*^ OR “number sense”) AND (“home numeracy” OR “home learning” OR “parent talk” OR parent^*^ OR SES^*^ OR socio^*^) AND (math^*^ OR arithmet^*^ OR calculation OR performance). This yielded 714 hits in total (530 from Web of Science and 184 from ERIC). From these, 62 articles were overlapping and therefore eliminated. Consequently, we ended up with 652 articles for abstract screening.

### Abstract Screening

The abstracts were read and evaluated by three independent raters: The first author of this study screened all the abstracts, in combination with the second and the fourth authors who shared and screened the abstracts in halves. In other words, every abstract was read by two raters. The raters scored the relevancy for the review on a three-point scale: 1 = “*relevant*” if home numeracy (i.e., frequency of activities and talk, but not parents' expectations, attitudes, or SES) was measured as the independent variable and children's mathematical skills were examined as the dependent variable (*n* = 74); 2 = “*maybe relevant*” if the assessment of one of the independent or dependent variables was not clearly mentioned in the abstract (*n* = 71); 3 = “*not relevant*” if the study was not about home numeracy and mathematical skills at all (*n* = 507). Cohen's Kappa (κ) was calculated to determine the level of agreement between the raters (Cohen, 1960). This agreement was substantial, κ = 0.736, *p* < 0.001 (e.g., McHugh, 2012). The disagreements on the ratings of the articles were overcome via discussions. All papers that were categorized as “*related*” and “*maybe related*” (*n* = 145) were entered into the next phase of full-text screening.

### Full-Text Screening

In the full-text screening, all the articles were read in full by the first author and additional inclusion and exclusion criteria were applied: First, in the current study, “home numeracy” was defined as the parent–child interactions that include *activities* with numerical content. Therefore, the articles that operationalized “home numeracy” in another way (e.g., as parents' attitudes, expectations, perceived parental involvement, or family socio-demographics) were excluded (*n* = 34). Second, articles that did not measure any form of children's mathematical skills but tested for example parents' or teachers math skills were excluded (*n* = 23). Third, articles that reported only intervention but not pretest data were excluded (*n* = 22). Fourth, articles that did not present original, empirical data (e.g., reviews, project reports) were excluded (*n* = 15). Fifth, articles of which the full text could not be retrieved online were not included (*n* = 14). Hence, after full-text screening, 108 articles were additionally eliminated. As a result, 37 articles were analyzed in this review.

## Results

**Figure 2:**
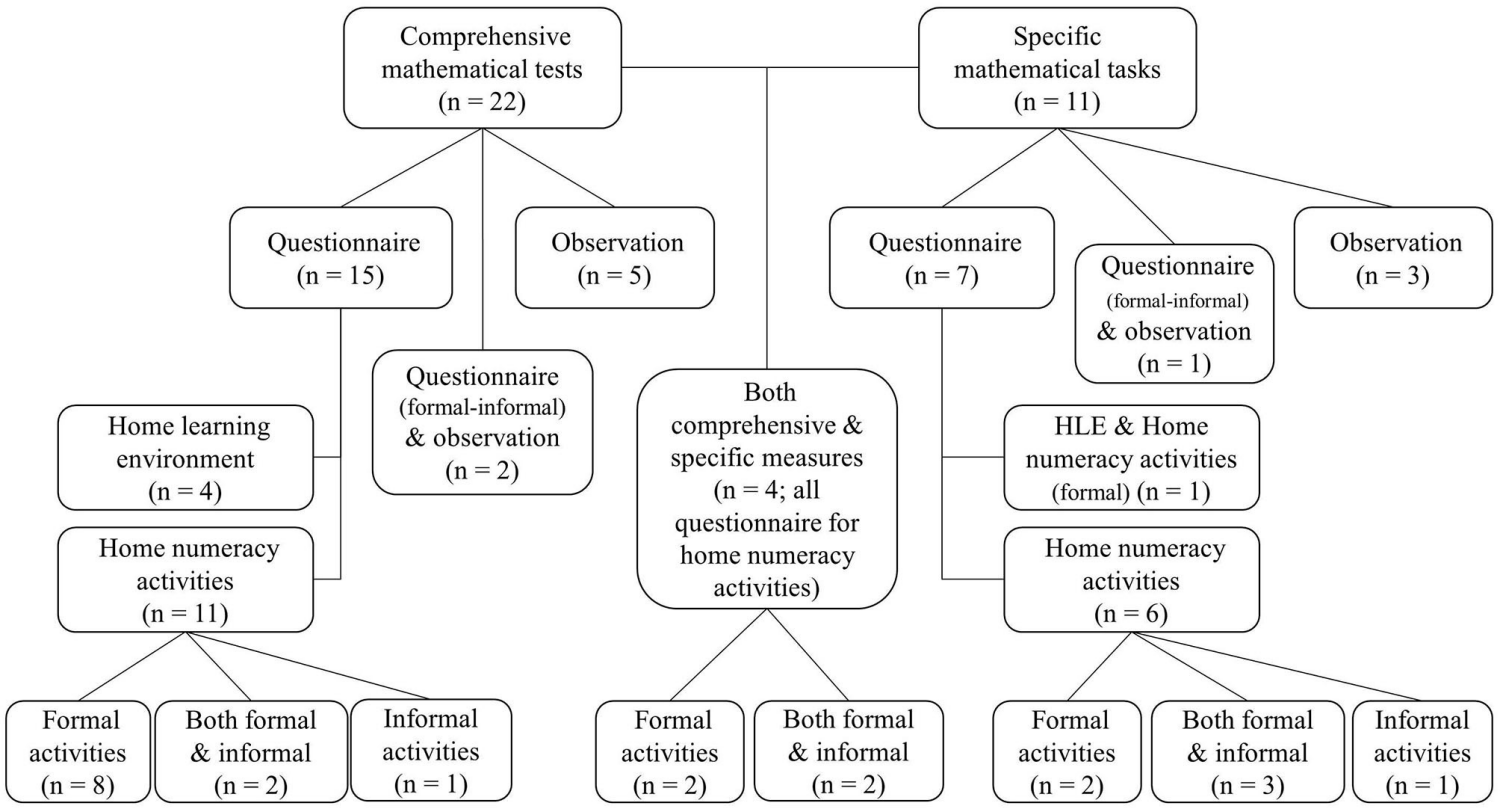
Summary and organizational chart of the reviewed articles.

Some commonalities were observed regarding the operationalization of both mathematical skills and home numeracy. Therefore, the studies were reviewed in two hierarchical levels (see Figure 2): At the higher level, we distinguished between the ways mathematical skills were operationalized, i.e., (A) comprehensive mathematical tests, which reveal a composite score from more than one task, (B) specific mathematical task(s), which reveal a single score of one task, or (C) both. At the next level, we distinguished between the methods used to evaluate home numeracy, i.e., (1) questionnaire, (2) observation, or (3) both, and elaborated on the assessment details. Finally, all the results were compiled and a *p*-curve analysis was run. Table 1 presents an overview with all the detailed information of the articles included in the review (e.g., sample characteristics, measures, and results).

**Table 1:** Detailed overview of the reviewed studies.

**References**	**Sample size and children's age in years**	**Sample location/ethnicity and SES**	**Home numeracy measure**	**Comprehensive mathematical tests**	**Specific mathematical tasks**	**Results**	**Recomputed *p*-values**
**Section A.1**							
Huntsinger et al. (1997)	*N:* 120 *M_*age*_:* 5.6	Ethnicity: Euro-American, Chinese-American, Taiwan-Chinese SES: high	Questionnaire: Formal activities	Test of Early Mathematics Ability (TEMA-2; Ginsburg and Baroody, 1990): Informal (relative magnitude, counting) and Formal (number facts, calculation, and base-ten concepts) mathematics subtests	–	Significant paths:Formal activities → composite math (β = 0.23^**^)	Not reported
Kleemans et al. (2012)	*N*: 89 *M_*age*_*: 6.1	Location:The NetherlandsSES: high	Questionnaire; Formal activities (adapted from LeFevre et al., 2009)	Utrecht Early Numeracy Test–Revised (Van Luit and Van de Rijt, 2009); comparison, linking quantities, correspondence, arranging, counting, estimation skills, knowledge of ordinal and cardinal aspects of number system, and ability to apply knowledge of the number system	–	Significant regressions:Formal activities → composite math(β = 0.33^*^)	*t*_(87)_ = 3.82, *p* = 0.00025
Segers et al. (2015)	*N:* 60 *M_*age*_*: 5.7	Location: The Netherlands SES: middle	Questionnaire; Formal activities (adapted from Kleemans et al., 2012)	Utrecht Early Numeracy Test-Revised	–	Significant regressions: Formal activities → composite math (β = 0.45^**^)	*t*_(50)_ = 3.53, *p* = 0.00090
Cheung et al. (2018)	*N:* 673 *M_*age*_*_:_ 4.3	Location: Philippines SES: low to middle	Questionnaire: Formal activities (adapted from LeFevre et al., 2009)	Composite math score from six tasks: numeral identification, object counting, rote counting, missing number, numerical magnitude comparison, addition	–	Significant result of interest: None	Not available
Anders et al. (2012)	*N:* 532 *M_*age*_:* T1: 3.7 T2: 4.7 T3: 5:7	Location: Germany SES: diverse	Questionnaire: Formal activities and interactions (from the Home Observation for Measurement of the Environment Inventory, HOME; Caldwell and Bradley, 1984, and Family Rating Scale; Kuger et al., 2005)	The German version of Kaufman Assessment Battery for Children (KABC; Melchers and Preuss, 2003): Counting, identifying numbers, knowledge of shapes, and addition and subtraction	–	Linear growth: Formal activities at T1 → composite math at T2 and T3 (*b* = 0.14^*^)	*t*_(530)_ = 2.8, *p* = 0.00530
Napoli and Purpura (2018)	*N:* 114 *M_*age*_*: T1: 4.1 T2: 4.6	Location: USA SES: middle to high	Questionnaire; Formal activities (adapted from LeFevre et al., 2009) at T1	Preschool Early Numeracy Skills Screener–Brief version (PENS-B; Purpura et al., 2015); Comparison, one-to-one correspondence, and ordinarily	–	Significant correlations: Formal activities at T1 and composite math at T2 (*r* = 0.40^***^)	*r*_(112)_ = 0.40, *p* = 0.00001
Del Rio et al. (2017)	*N:* 180 (mothers & fathers) *M_*age*_*: 5.6	Location: Chile SES: low and high	Questionnaire; Advanced formal activities (adapted from Skwarchuk et al., 2014)	Applied Problems subset of the Woodcock–Munoz Bateria III (Muñoz-Sandoval et al., 2005); Orally and visually presented numeration and calculations	–	Significant correlations:Advanced formal activities of mothers and composite math(*r* = 0.21^**^)	*r*_(178)_ = 0.21, *p* = 0.00466
Zippert and Ramani (2017)	*N:* 43 *M_*age*_*: 4.5	Location: USA SES: middle to high	Questionnaire; Basic and advanced formal activities	Conventional math; rote counting, counting principles, numeral identification Non-symbolic math; non-symbolic magnitude comparison and addition Advanced math; symbolic magnitude comparison and arithmetic	–	Significant correlations: Advanced formal activities and composite advanced math (*r* = 0.41^**^)	*r*_(41)_ = 0.41, *p* = 0.00632
Niklas and Schneider (2014)	*N:* 340 *M_*age*_:* T1: 4.8 T2: 6.1 T3: beginning of grade 1 T4: end of grade 1	Location: Germany SES: diverse	Questionnaire: Informal (game) activities at T2	Math battery at T1 and T2 (Krajewski, 2005): counting, naming numbers, solving simple calculations, matching, comparing quantities Performance Indicators in Primary School (PIPS; Tymms and Albone, 2002): calculations, naming 2–3-digit numbers, geometry at T3 “Deutscher Mathematiktest fur erste Klassen” (DEMAT 1+; Krajewski et al., 2002) solving written math problems, linking numbers to quantities	–	Structural Equation Modeling: Informal activities → composite math at T4 (β = 0.18^*^)	*r*_(338)_ = 0.15, *p* = 0.00558
Ciping et al. (2015)	*N:* 177 *M_*age*_*: T1: 6.7 T2: 7.7	Location: China SES: diverse	Questionnaire; Formal and informal activities (adapted from LeFevre et al., 2009) at T1 and T2	Composite score of calculation fluency and Omitted number task at T1 and T2	–	Significant paths: Composite math at T1 → formal activities at T2 (β = −0.15^*^)	*r*_(175)_ = −0.18, *p* = 0.01651
Huntsinger et al. (2016)	*N:* 97 *M_*age*_*: T1: 4.9 T2: 5.8	Location: USA SES: middle	Questionnaire: Formal and informal activities at T1	TEMA-2: Formal and informal subtests at T2	–	Significant regressions: Formal activities at T1 → composite math at T2 (β = 0.13^**^)	*z* = 2.43, *p* = 0.01510
Baker (2015)	*N:* 1,202 (boys and mothers) *M_*age*_*: 5.6	Ethnicity: African-American SES: diverse	Questionnaire: Home learning environment (HLE) (adapted from LeFevre et al., 2009)	Math test battery: Understanding of numbers, geometry, and spatial relations	–	Significant regressions: HLE → composite math (β = 0.06^*^)	*r*_(1, 200)_ = 0.08, *p* = 0.00552
Melhuish et al. (2008)	*N:* 2,354 *M_*age*_*: T1: 3 T2: 5 T3: 7	Location: UK SES: diverse	Questionnaire: Home learning environment (HLE) at T1	Early Number Concepts subscale of the British Ability Scales (BAS II; Elliot et al., 1996) at T2: *details not provided* Nationally standardized math achievement test at T3	–	Significant regressions: HLE → composite math at T2 (effect size = 0.65) and T3 (effect size = 0.50)	Not reported
Niklas and Schneider (2017)	*N:* 434 *M_*age*_:* T1: 4.8 T2: end of grade 1 T3: middle of grade 4	Location: Germany SES: low to medium	Questionnaire: Home learning environment (HLE) at T1	Math battery (Krajewski, 2005) at T1 DEMAT 1+ at T2 Standardized and curriculum-based test KLASSE 4 (Lenhard et al., 2011): geometrical and written math problems at T3	–	Significant regressions: HLE → composite math at T3 (β = 0.72^**^)	*t*_(432)_ = 2.98, *p* = 0.00305
Visser et al. (2019)	*N*: 10376 *M_*age*_*: T1: pre-Grade 1 T2: Grade 5	Location: South Africa SES: diverse	Questionnaire: Home learning environment (HLE): reading–writing, playing games, songs–stories at T1	Math Achievement test at T2	–	Significant regressions:Game-based HLE at T1 → Math skills at T2 (β = 0.17^***^)	*r*_(10, 374)_ = 0.19, *p* ≤ 0.00001
**Section A.2**							
Elliott et al. (2017)	*N:* 54 (mothers) *M_*age*_*: 5.8	Location: USA SES: high	Observation: Semi-structured play for 10 min with toys, such as books, kitchen tools, puppets, cash register	Form A of TEMA-3: Verbal counting, comparison, numeral literacy, number facts, calculation, number concepts	–	Significant regressions: Maternal advanced math talk → composite math (β = 0.42^*^)	*r*_(52)_ = 0.39, *p* =0.00355
Leyva (2019)	*N*: 210 *M*_age_: T1: beginning of prekindergarten T2: end of kindergarten	Location: Chile SES: low	Observation: Parent–child interaction during grocery game play at T1	Applied Problems subset of the Woodcock–Muñoz Bateria III (among other skills, counting, comparing quantities, adding, and/or subtracting) at T2	–	Significant relations: Math support at T1 → Math skills at T2 (*d* = 0.13)	Not reported
Susperreguy and Davis-Kean (2016)	*N:* 40 (mothers) *M_*age*_:* T1: 4.5 T2: 5.6	Location: USA SES: diverse	Observation: Unstructured parent–child interaction during meal times for 2 days at T1	TEMA-3 at T2	–	Significant regressions: Maternal math talk → composite math (β = 0.31^*^)	*t*_(31)_ = 2.18, *p* = 0.03697
Leyva et al. (2019)	*N*: 208 *M_*age*_*: 6.6 1st grade	Ethnicity: Chinese, Mexican African-American, and Dominican living in the USA SES: low	Observation: Parent–child interaction during grocery game play	Woodcock–Johnson III Tests of Achievement: number calculation (counting, adding, subtracting, and comparing quantities) and math concepts (understanding whole-number place value)	–	Significant result of interest: None	Not available
Zhou et al. (2006)	*N:* 85 *M_*age*_:* 4	Location: China SES diverse:	Observation: Semi-structured activities for 15 min each, book reading, paper activity, worksheet, blocks	Composite score of three versions of give-a-number task	–	Significant result of interest: None	Not available
**Section A.3**							
Lehrl et al. (2019)	*N:* 229 *M_*age*_:* T1: 3 T2: 4 T3: 5 T5: 12	Location: Germany SES: diverse	Questionnaire: Formal activities Observation: Semi-structured book reading at T1, T2, and T3	Numerical skills: subscale “arithmetic” of the German Version of the Kaufman Assessment Battery for Children (K-ABC) at T3 Mathematics: content-related subtest (quantity, space and shape, change and relationship, data and change) and Process-related subtest (applying technical skills, modeling, arguing, communicating, representing, problem solving) at T5	–	Significant regressions: Math talk → mathematics at T5 (β = 0.13^*^)	*r*_(227)_ = 0.20, *p* = 0.00236
Ramani et al. (2015)	*N:* 33 *M_*age*_*: 4.3	Ethnicity: Caucasian, African-American, Hispanic living in the USA SES: low	Observation: Semi-structured parent–child interaction during book reading, puzzle making, and board game playing Questionnaire: Formal and informal activities (adapted from LeFevre et al., 2009)	Composite score of basic math skills: verbal counting and number identification Advanced math skills: counting principles, enumeration and cardinality, number line estimation and comparison	–	Significant regressions: Formal activities → basic composite math (β = 0.34^*^) Advanced math talk → advanced composite math skills (β = 0.33^*^)	*r*_(31)_ = 0.55, *p* = 0.00091
**Section B.1**							
Dearing et al. (2012)	*N:* 127 *M_*age*_*: 6.7	Ethnicity: Latino, Asian, African-American and White living in the USA SES: diverse	Questionnaire: Formal activities (adapted from LeFevre et al., 2009) and home learning environment (HLE)	–	Calculations: addition and subtraction	Structural equation models: HLE → calculations (*r* = 0.19^*^) Formal activities → calculations (*r* = 0.29^*^)	*r*_(125)_ = 0.19, *p* = 0.03239
Kleemans et al. (2013)	*N:* 150 *M_*age*_*: T1: 6 at kindergarten T2: 7 at 1st grade	Location: The Netherlands SES: middle to high	Questionnaire: Formal activities → (adapted from LeFevre et al., 2009) at T1	–	Calculations: addition and subtraction at T2	Significant regressions:Formal activities → addition (β = 0.24^***^)Formal activities → subtraction(β = 0.23^**^)	*r*_(148)_ = 0.63, *p* <0.00001
Kleemans et al. (2018)	*N:* 103 *M_*age*_*: T1: 6 at kindergarten T3: 8 at 2nd grade	Location: The Netherlands SES: middle to high	Questionnaire: Formal activities (adapted from LeFevre et al., 2009) at T1	–	Calculations: addition and subtraction with small and large numbers at T3	Significant regression: Formal activities → large numbers arithmetic (β = 0.22^***^)	*r*_(101)_ = 0.41, *p* = 0.00002
Huang et al. (2017)	*N:* 104 (mothers & fathers) *M_*age*_*: 5	Location: China SES: N/A	Questionnaire: Formal and informal activities (adapted from LeFevre et al., 2009)	–	Calculations: Verbal story problems and written arithmetic problems	Significant regressions: Mothers number practices (β = 0.32^*^) and number book activities (β = −0.31^*^) → story problems Mothers' number practices (β = 0.35^*^) and fathers' games (β = 0.29^*^) and applications (β = 0.30^*^) → written arithmetic	*r*_(100)_ = 0.20, *p* = 0.04386
Benavides-Varela et al. (2016)	*N*: 110 *M_*age*_*: 5.9	Location: Italy SES: diverse	Questionnaire: Informal activities (playing board game)	–	Five tasks: Counting, one-to-one correspondence, magnitude comparison, number line task, everyday numerical problems	Significant correlations: Informal activities → counting (*r* = 0.31^*^)	*r*_(108)_ = 0.31, *p* = 0.00098
Mutaf-Yildiz et al. (2018a)	*N*: 128 *M_*age*_*: 5.4	Location: Belgium SES: middle to high	Questionnaire: Formal and informal activities (adapted from LeFevre et al., 2009)	–	Basic number skills: Non-symbolic and symbolic comparison Non-symbolic and symbolic number line estimation Enumeration and connecting Pictorial and symbolic calculations	Significant regressions: Formal activities → enumeration (β = 0.21^*^) Informal activities → symbolic number line (β = −0.18^*^) Informal activities → pictorial calculation (β = 0.17^*^)	*r*_(126)_ = 0.21, *p* = 0.01735
Vasilyeva et al. (2018)	*N:* 98 *Mage:* T1: 5.8 T2: 6.8	Location: Russia SES: N/A	Questionnaire: Formal and informal activities at T1	–	4 tasks: Raven's test, number identification, numerical magnitude comparison, arithmetic at T2	Significant paths: Formal activities → number identification (effect size = 0.42^**^) Informal activities → magnitude comparison (effect size = 0.37^*^) Both activities → arithmetic (effect sizes = 0.30^*^ and.39^**^)	*r*_(96)_ = 0.41, *p* = 0.00003
**Section B.2**							
Levine et al. (2010)	*N:* 44 *M_*age*_:* (in months) T1: 14 m T2: 18 m T3: 22 m T4: 26 m T5: 30 m T6: 46 m	Location: USA SES: diverse	Observation: Unstructured activity sessions at T1, T2, T3, T4, and T5	–	Point-to-X-task at T6	Significant regressions: Number talk → point-to-X (β = 0.29^*^)	*r*_(42)_ = 0.47, *p* = 0.00129
Gunderson and Levine (2011)	*N:* 44 *M_*age*_:* (in months) T1: 14 m T2: 18 m T3: 22 m T4: 26 m T5: 30 m T6: 46 m	Location: USA SES: diverse	Observation: Unstructured activity sessions at T1, T2, T3, T4, and T5	–	Point-to-X-task at T6	Significant regressions: Number talk with present objects in large sets (4–10) → point-to-X (β = 0.38^*^)	Not reported
Glenn et al. (2018)	*N:* 60 *M_*age*_:* T1: 2 T2: 3.5	Location: USA SES: diverse	Observation: Unstructured activity sessions at home for 90 min at T1	–	Point-to-X-task at T2	Significant random effect intercept: Number talk → point-to-X (random effects intercept: 3.95^***^)	Not reported
**Section B.3**							
Mutaf-Yildiz et al. (2018b)	*N*: 44 *M_*age*_*: 5.6	Location: Belgium SES: middle to high	Questionnaire: Formal and informal activities (adapted from LeFevre et al., 2009) Observations: Semi-structured activities for 5 min each, book reading and Lego building	–	Calculations: Addition and subtraction	Significant correlations: Formal activities → calculation (*r* = 0.31^*^) Parent number talk → calculation (*r* = −0.35^*^)	*r*_(42)_ = 0.31, *p* = 0.04057
**Section C**							
LeFevre et al. (2009)	*N*: 146 *M_*age*_*: 6.5	Location: Canada SES: diverse	Questionnaire: Formal and informal activities	KeyMath Test–Revised Form B (Connolly, 2000): The Numeration subtest: “math concepts and number system knowledge,” including quantity, digit recognition, place value The addition and subtraction subtests	Calculation fluency (single-digit addition)	Significant regression: Game activities → composite math (β = 0.18^*^) Number skills (β = 0.21^*^) games (β = 0.21^*^), and application activities (β = 0.24^*^) → math fluency	*r*_(144)_ = 0.27, *p* = 0.00098
Manolitsis et al. (2013)	*N:* 82 *M_*age*_:* 5.4 T1 beginning of KG T2 end of KG T3 end of 1st grade	Location: Greece SES: diverse	Questionnaire: Formal activities (adapted from LeFevre et al., 2009)	TEMA-3: Cardinality rule, seriation of numbers, naming of single-digit numbers, and number comparison at T1 and T2	Counting at T1 and 2 Math fluency at T3	Significant correlations: Formal activities and counting at T1 (*r* = 0.28^*^)	*r*_(80)_ = 0.28, *p* = 0.01084
Skwarchuk et al. (2014)	*N:* 121 *M_*age*_*: 5.8	Location: Canada SES: diverse	Questionnaire: Formal activities and informal home numeracy (knowledge of commercially available number games)	The Numeration subtest of Key Math–Revised	Non-symbolic arithmetic	Significant regressions: Informal activities → non-symbolic arithmetic (β = 0.20^*^) Advanced formal activities → math (β = 0.21^*^)	*r*_(119)_ = 0.30, *p* = 0.00083
Missall et al. (2015)	*N:* 72 *M_*age*_*: 4.4	Ethnicity: White, Hispanic, Asian, multiracial living in the USA SES: diverse	Questionnaire: Numeracy activities	The quantitative subtest and the School Readiness Composite of Bracken Basic Concepts Scale—Third edition: Receptive (BBCS-3:R; Bracken, 2006)	The Individual Growth and Development Indicators of Early Numeracy (IGDIs-EN; Hojnoski and Floyd, 2013); One-to-one correspondence, verbal counting, number naming, and quantity comparison	Significant correlations: None Significant regressions: None	Not available

* *p < 0.05*,

** p < 0.01, and

*** *p < 0.001*.

### A. The Relationship Between Home Numeracy and Children's Performance as Measured by Comprehensive Mathematical Tests

#### A.1. Home Numeracy Operationalized With Questionnaires

In total, 22 studies examined the relationship between home numeracy and comprehensive mathematical tests. Fifteen of these studies used questionnaires to assess home numeracy. Most of these questionnaires were inspired by the work of LeFevre et al. (2009), specifically targeting *numeracy activities* (*n* = 11). A minority of the studies assessed the broader *home learning environment* of which home numeracy is a part (*n* = 4).

Of the 11 studies that specifically addressed numeracy activities, 8 investigated the effects of *formal* home numeracy activities on mathematical skills. Huntsinger et al. (1997) recruited families from different ethnic backgrounds with a high socio-economic status and showed that children (*M*_*age*_ = 5.5 years) with a higher composite mathematical score (i.e., TEMA-2) were the ones whose parents spent more time in formal teaching practices, such as helping with mathematics homework, regardless of the ethnicity. They also showed however that Chinese-American and Taiwan-Chinese parents engaged more in formal activities than Euro-American parents and that this higher engagement was associated with higher mathematics scores in Chinese-American and Taiwan-Chinese children. Two studies with participants from middle- to high-SES families showed that formal numeracy activities were positively associated with children's (*M*_*age*_ = 6 years) composite mathematical score (i.e., Utrecht Early Numeracy Test-Revised) above and beyond children's cognitive skills, linguistic skills, and their home literacy environment (Kleemans et al., 2012; Segers et al., 2015). However, one study with participants from low- to middle-SES families reported that children's (*M*_*age*_ = 4.3 years) composite mathematical scores were not associated with formal home numeracy activities, but positively related with SES (Cheung et al., 2018). Next to these cross-sectional designs, several studies used a longitudinal approach. Anders et al. (2012) found that both families' SES and formal home numeracy activities were associated with kindergartner's (*M*_*age*_ = 3.08) concurrent mathematical score and also its growth in the following 2 years. Another study showed that in a group of middle- to high-SES participants formal numeracy activities were the most stable (positive) predictors of kindergartners' (*M*_*age*_ = 4.09 years) composite mathematical score one semester later, even after accounting for control variables, such as a child's age, gender, and parental education (Napoli and Purpura, 2018).

Two studies explicitly distinguished between basic and advanced formal numeracy activities (Del Rio et al., 2017; Zippert and Ramani, 2017). In a group of middle- to high-SES American participants advanced formal number activities (e.g., solving simple sums), but not basic ones (e.g., counting), were positively related with children's (*M*_*age*_ = 4.5 years) performance in composite score of advanced (i.e., symbolic comparison and arithmetic) mathematical skills (Zippert and Ramani, 2017). The other study recruited Chilean families from low and high socio-economic backgrounds and assessed mothers' and fathers' home numeracy separately (Del Rio et al., 2017). These authors found that children's (*M*_*age*_ = 5.6 years) composite mathematical score (i.e., Applied Problems subtest of the Woodcock–Muñoz Batería III) was positively associated with SES and mothers' advanced formal activities at home, but not with the ones of the fathers. However, the relationship between SES and mothers' advanced home numeracy activities was negative.

Niklas and Schneider (2014) recruited participants from diverse socio-economic backgrounds in Germany and assessed *only informal* activities, the authors reported that the frequency of playing games at the end of kindergarten (*M*_*age*_ = 6.4 years) positively predicted children's mathematical composite score at the end of Grade 1 even after controlling for SES. Furthermore, similar to Del Rio et al. (2017), it was found that SES was negatively related to informal activities whereas it was positively associated with children's mathematical composite score. However, two studies that measured *both formal* and *informal* home numeracy activities with a longitudinal approach (Ciping et al., 2015; Huntsinger et al., 2016) reported conflicting results. Huntsinger et al. (2016) showed that in a group of middle- to high-SES American families, formal numeracy activities at the mean age of 4.48 years were the most consistent (positive) predictor of kindergartners' composite mathematical score (TEMA-2) 1 year later (*M*_*age*_ = 5.6 years), but informal activities were not. Ciping et al. (2015) took another approach and investigated the effect of children's mathematical skills on home numeracy in a group of Chinese participants with diverse socio-economic backgrounds. These authors observed that the composite mathematical score of two tasks (i.e., calculation fluency and omitted number) at Grade 1 (*M*_*age*_ = 6.7 years) negatively predicted the formal, but not the informal numeracy activities at Grade 2. Furthermore, no moderation effect from SES was found.

In addition to studies that focused on home numeracy activities, four studies investigated the relation between the general *home learning environment* (HLE) and mathematical skills. Baker (2015) demonstrated in a sample of African-Americans from diverse socio-economic backgrounds that boys' (*M*_*age*_ = 5.6 years) composite mathematical score (i.e., National Assessment of Educational Progress) were positively associated with SES and also with mothers' home learning activities even after controlling for children's age and family demographics (e.g., income, maternal age, and education). Three other studies investigated the longitudinal associations of HLE on mathematics (Melhuish et al., 2008; Niklas and Schneider, 2017; Visser et al., 2019). Home learning environment of participants in the UK from diverse socio-economic backgrounds, assessed at age 3, was the strongest predictor of children's composite mathematical score (i.e., the Early Number Concepts subscale of the British Ability Scale II) both at ages 5 and 7, over and above the effects of maternal education and social class (Melhuish et al., 2008). Niklas and Schneider (2017) recruited families from low- to middle-SES in Germany and found that the composite mathematical scores at the middle of Grade 4 were positively predicted by the home learning environment assessed at kindergarten, even when earlier mathematical skills, other child (i.e., age, sex, and intelligence) and family characteristics (i.e., socio-economic status and migration background) were accounted for. Families' SES was not a direct predictor of composite mathematical scores; however, SES was associated with HLE. Similar findings were reported by Visser et al. (2019) in a sample of South African families from diverse socio-economic backgrounds. Home learning environment measured in kindergarten was positively associated with Grade 5 children's standardized mathematical scores, even after accounting for earlier mathematical skills and SES.

#### A.2. Home Numeracy Operationalized With Observations

Five studies used observations to index home numeracy, and two of them focused on mothers only. Elliott et al. (2017) observed that highly educated American mothers' numeracy talk of large numbers (>10) was positively associated with children's (*M*_*age*_ = 5.7 years) composite mathematical score (i.e., TEMA-3), whereas this was not true for talk about small (1–5) and medium (6–10) numbers, or overall number talk. Two longitudinal studies showed that the frequency of numeracy talk was predictive for children's mathematical skills (Susperreguy and Davis-Kean, 2016; Leyva, 2019). Leyva (2019) showed that low-income Chilean parents' math talk when children were in the beginning of pre-kindergarten (*M*_*age*_ = 4.5 years) was predictive for children's composite mathematical scores (Applied Problems subset of the Woodcock-Muñoz Bateria III) at the end of kindergarten, even after controlling for age, earlier mathematical skills, and parents' education. In a sample of children from diverse socio-economic backgrounds, Susperreguy and Davis-Kean (2016) found that American mothers' numeracy talk with their children (*M*_*age*_ = 4.5 years) was predictive for children's composite mathematical scores (TEMA-3) 1 year after the recordings, even after controlling for maternal education and children's self-regulation (i.e., inhibitory control) and working memory. By contrast, two studies reported the absence of a relationship between numeracy talk and mathematical skills (Zhou et al., 2006; Leyva et al., 2019). Leyva et al. showed that the frequency of numeracy talk in low-income families with diverse ethnic backgrounds from the USA did not relate to children's (*M*_*age*_ = 6.6 years) composite math scores (Woodcock-Johnson III Tests of Achievement, WJ-III; Woodcock et al., 2001). Ethnicity or mothers' education did not have any effect on children's mathematical skills either. Zhou et al. (2006) found that the frequency of numeracy talk in Chinese families from diverse socio-economic background was equal in “high” and “low” math achievers (*M*_*age*_ = 4.1 years).

#### A.3. Home Numeracy Operationalized With Both Questionnaires and Observations

There are two studies that used both a questionnaire and semi-structured observations to assess the frequency of numeracy activities and the frequency of numeracy talk, respectively (Ramani et al., 2015; Lehrl et al., 2019). Ramani et al. (2015) recruited children and their parents from Head Start centers (low-SES) in the USA and found that children's (*M*_*age*_ = 4.3 years) composite score of basic mathematical skills (i.e., verbal counting and numeral identification) was positively associated with formal home numeracy activities as indexed by the questionnaire, but not with the observed basic (i.e., counting and identifying numbers) or advanced (i.e., labeling number of elements in a set, ordering numbers, and arithmetic) numeracy talk. By contrast, the composite score of advanced mathematical skills (i.e., counting principles, enumeration and cardinality, number line estimation, and numeral magnitude comparison) was positively related with the observed advanced numeracy talk—but not with basic talk or formal activities. Lehrl et al. (2019) investigated the longitudinal associations between home numeracy and mathematics in participants from Germany from diverse socio-economic backgrounds and showed that numeracy talk, but not home numeracy activities, at ages 3, 4, and 5, positively predicted children's mathematical achievement at age 12 even when SES was controlled for.

### B. The Relationship Between Home Numeracy and Children's Performance on Specific Mathematical Tasks

#### B.1. Home Numeracy Operationalized With Questionnaires

Eleven studies used specific tasks to examine children's mathematical skills. Seven of them made use of questionnaires, of which six specifically focused on home numeracy *activities* and one on both numeracy *activities* and the broader concept of *home learning environment*.

Results from a sample with children from diverse socio-economic backgrounds living in the USA showed that SES was positively related with the HLE. In turn, both the HLE and formal home numeracy activities were positively associated with girls' (*M*_*age*_ = 6.7 years) addition and subtraction skills (Dearing et al., 2012). Kleemans et al. investigated the long-term effects of formal numeracy activities on children's calculation skills in families from middle- to high-SES living in the Netherlands (Kleemans et al., 2013, 2018). Results showed that formal numeracy activities when children were in kindergarten (*M*_*age*_ = 6 years) positively predicted children's addition and subtraction scores 1 year later in first grade, even when cognitive and linguistic child factors were controlled for (Kleemans et al., 2013). Following the same children, Kleemans et al. (2018) found that formal home numeracy activities positively predicted children's arithmetic performance with large but not small problem sizes 2 years later (Kleemans et al., 2018). Huang et al. (2017) assessed mothers and fathers from China separately and found that children's (*M*_*age*_ = 5 years) word problem solving skills (e.g., “Emma has four pens. Her sister gives two more. How many pens does Emma have now?”) were positively correlated with mothers' formal number practices and negatively with mothers' number book activities. Symbolic calculation (e.g., 1 + 3) scores were positively related with mothers' number practices and fathers' informal (games and application) activities. There are three studies that showed that home numeracy is differentially related with specific mathematical tasks (Benavides-Varela et al., 2016; Mutaf-Yildiz et al., 2018a; Vasilyeva et al., 2018). In a sample of Italian families from diverse socio-economic backgrounds, Benavides-Varela et al. (2016) found that counting skills in children (*M*_*age*_ = 5.9 years), but not one-to-one correspondence, everyday numerical problems, non-symbolic magnitude comparison, and number line estimation, were positively correlated with the frequency of playing board games, but not with any other type of home numeracy activities, such as playing videogames or reading book even after controlling for SES. Mutaf-Yildiz et al. (2018a) reported that formal home numeracy activities in Belgian families from middle- to high-SES backgrounds were positively associated with children's (*M*_*age*_ = 5.4 years) enumeration (i.e., sequentially tapping the correct number of dots indicated by a digit), but not with symbolic and non-symbolic comparison or number line estimation tasks, and not with a non-symbolic symbolic connecting task (i.e., choosing a set of dots equivalent to a target digit) or pictorial calculations even after taking SES into account. This study further demonstrated that informal home numeracy was positively associated with pictorial calculations and performance on a symbolic number line estimation task, but not with the other tasks. Finally, in a longitudinal study, Vasilyeva et al. (2018) observed that Russian families' formal home numeracy activities, at age 5.8 on average, positively predicted children's number identification skills and informal activities positively predicted symbolic comparison skills, whereas both types of activities were associated with arithmetic performance 1 year later.

#### B.2. Home Numeracy Operationalized With Observations

Three longitudinal studies used observations to index home numeracy and examined its association with specific numerical skills in families from the US with diverse socio-economic backgrounds. Levine et al. (2010) administered the Point-to-X task, i.e., a task in which participants have to indicate which of the two pictures contains a certain number of items (Wynn, 1992) to measure the cardinal-principle knowledge of 3.8-year-old children. Parent–child dyads were observed in an unstructured way during five home visits, each lasting 90 min. Especially parents' number talk positively predicted children's cardinal-principle knowledge, even after controlling for SES (education level of the primary caregiver who interacted with the child during the observations and income) and for parents' total amount of talk (Levine et al., 2010; see also Glenn et al., 2018). A more detailed analysis (Gunderson and Levine, 2011) of the same data revealed that specifically the talk about large sets (4–10) of present objects positively predicted the children's score on a Point-to-X task, even after controlling for SES and other types of parental numeracy and non-numeracy talk. In this sample, SES was positively related with parents' number talk which in turn was positively associated with children's mathematical skills.

#### B.3. Home Numeracy Operationalized With Both Questionnaires and Observations

Only one study used both a questionnaire and semi-structured observations to measure home numeracy in Belgian families from middle to high socio-economic backgrounds (Mutaf-Yildiz et al., 2018b). Results showed that home numeracy assessed with a questionnaire on the one hand and with observations on the other were not correlated with each other. Moreover, this study showed that children's (*M*_*age*_ = 5.6 years) calculation skills positively correlated with the frequency of formal home numeracy activities measured with a questionnaire, whereas these calculation skills negatively correlated with parents' observed numeracy talk (Mutaf-Yildiz et al., 2018b).

### C. The Relation Between Home Numeracy and Children's Performance in Both Comprehensive Mathematical Tests and Specific Mathematical Tasks in One Study

In four studies, home numeracy was indexed with questionnaires and children's mathematical skills were examined with both a composite score and specific mathematical measures (LeFevre et al., 2009; Manolitsis et al., 2013; Skwarchuk et al., 2014; Missall et al., 2015). These studies revealed conflicting results. In a Canadian sample of children from diverse socio-economic backgrounds, LeFevre et al. (2009) found that a composite mathematical score (i.e., KeyMath–Revised) was positively associated with informal activities (games) over and above the effects of children's (*M*_*age*_ = 6.5 years) vocabulary, spatial memory span, and SES. On the other hand, in another similar Canadian sample, Skwarchuk et al. (2014) observed that children's (*M*_*age*_ = 5.8 years) composite mathematical score (i.e., KeyMath–Revised) was not associated with informal home numeracy or basic formal activities, but that it was only positively related with advanced formal practices even after accounting for SES. In this study, SES also positively correlated with mathematical skills. Moreover, LeFevre et al. (2009) showed that a specific measure of calculation fluency (solving problems correctly as fast as possible) was positively related to both formal and informal activities, even when SES was accounted for, whereas Manolitsis et al. (2013) reported that formal number activities in Greek families from diverse socio-economic backgrounds were only positively correlated with specific verbal counting skills (counting from 1 to highest number children could) of children (*M*_*age*_ = 5.4 years), but not with math fluency or a composite mathematical score (i.e., TEMA-3). In this study, SES was not related with home numeracy activities or mathematical skills. However, Skwarchuk et al. (2014) reported that a specific measure of non-symbolic calculation was positively associated with informal home numeracy (i.e., parents' number game knowledge) but not with formal activities, even after controlling for family income. By contrast, Missall et al. (2015) recruited families from diverse ethnicities and SES living in the USA and documented that home numeracy—which was calculated as a sum score of both formal and informal activities—did not predict any type of (composite or specific) mathematical skill in children (*M*_*age*_ = 4.4 years). The authors also reported that neither ethnicity nor income were related to home numeracy activities.

### *P*-Curve Analysis

It became clear that 33 studies out of 37 observed significant associations between home numeracy and children's mathematical skills. However, one should remember that there is a bias to publish studies with positive findings compared with the ones with negative findings (e.g., Joober et al., 2012). Therefore, we tested whether these significant (*p* < 0.05) relations were the result of a publication bias, or whether they indicate a true effect, by means of a *p*-curve analysis (Simonsohn et al., 2013, 2014). A *p*-curve analysis answers this possibility of a publication bias by producing a distribution of statistically significant *p*-values for a set of studies. Simonsohn et al. (2014) reasoned that a right-skewed *p*-curve would be generated if there is a true effect because more low (e.g., *p* < 0.01) significant *p*-values are expected than high significant *p*-values (e.g., *p* = 0.04) in a set of studies. On the contrary, if the significant relations in a set of studies were the result of a publication bias, a left-skewed *p*-curve would be revealed because more high *p*-values (e.g., *p* = 0.05) and less low *p*-values (e.g., *p* < 0.01) would be observed in such a set of studies.

Simonsohn et al. (2014) developed an online application “*p*-curve app 4.06” (http://www.p-curve.com/app) to generate a *p*-curve. The app uses available test statistics (*r, t, z, f*, *F*, or χ^2^) from each study, along with the degrees of freedom, and recalculates the *p*-values (see the last column of Table 1). Because the *p*-curve analysis assumes that all the *p*-values that are to be analyzed are statistically independent from each other, including only one statistical test from each reviewed paper in the analysis is advised. Consequently, in the papers where two or more results were reported, a decision must be made to select only one of them. To keep this choice objective, we followed Simonsohn's (2014) recommendation and chose the result that was reported first in the manuscript to be included in our analysis. The *p*-curve analysis was run on only 28 papers because in four studies no significant relations were observed and in five other studies the required test statistics were not reported. Gunderson and Levine (2011) for instance documented a scatter plot displaying the relationship between home numeracy and mathematical skills, but did not report the actual effect size of the correlation.

**Figure 3:**
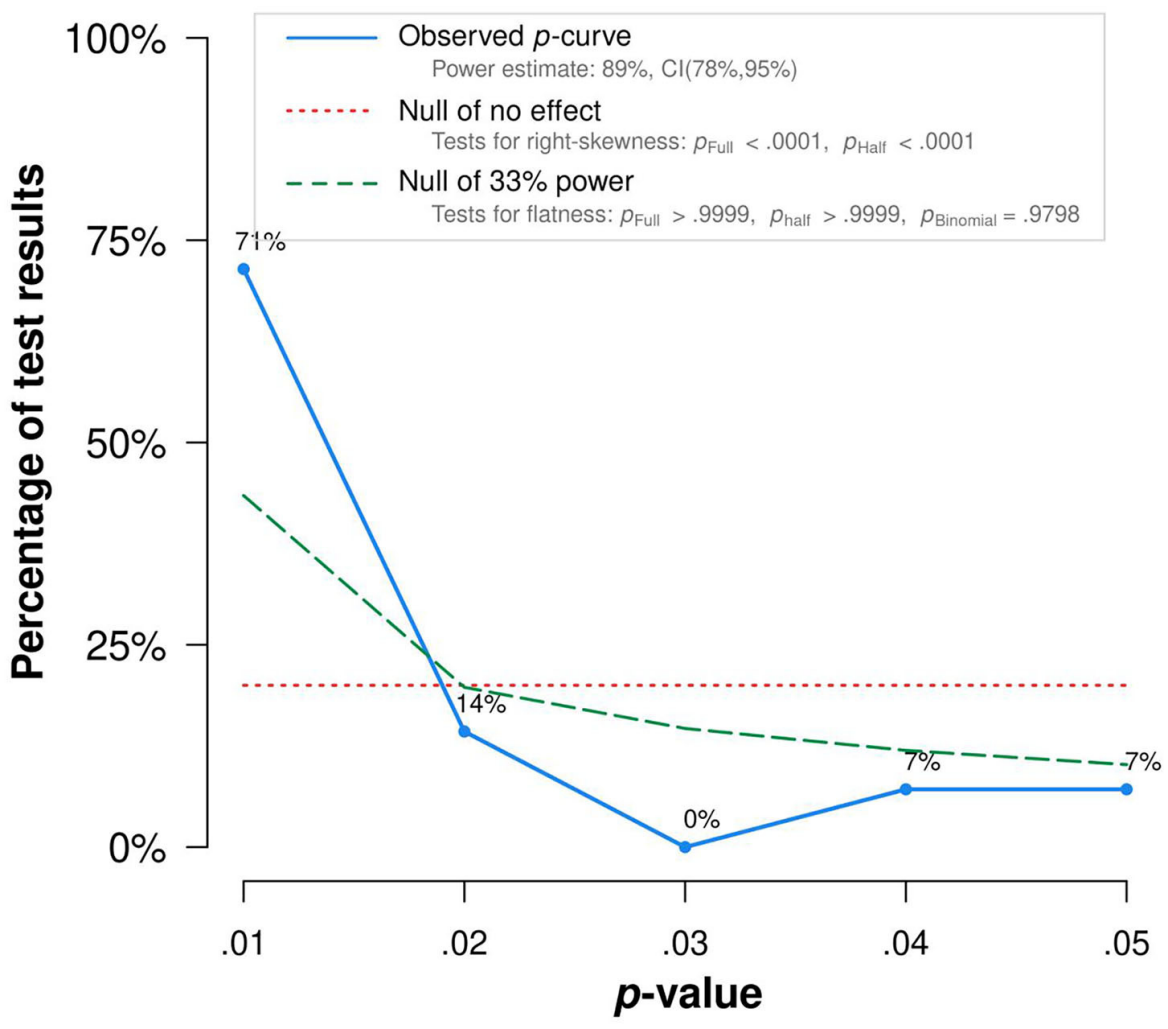
Results of the *p-*curve analysis.

Figure 3, revealed by the “p-curve app 4.06,” displays the results of the *p*-curve analysis. The two dashed lines are reference lines; the shorter-dashed line represents an expected distribution of *p*-values when there is no true effect and the longer-dashed line represents an expected distribution of *p*-values when there is a true effect. The observed *p*-curve of this set of studies, represented by the straight line, shows that there are more low (e.g.,0.01 s) *p*-values compared with higher (e.g.,0.04 s) ones, as we would expect when there is a true effect, with a high statistical power (89%). This result indicates that the positive relation between home numeracy and children's mathematical skills reported in the reviewed studies indeed reflects a true effect and therefore has evidential value.

## Discussion

The purpose of the current literature review was to provide a synthesized and systematic overview of the studies focusing on the relationship between home numeracy and children's mathematical skills. We observed that both concepts have been operationalized in a variety of ways. However, despite the use of diverse measures for home numeracy and mathematical skills, we found that 32 studies out of 37 revealed a positive association between home numeracy and children's mathematical skills. In addition, a *p*-curve analysis confirmed that this association reflects a true effect and cannot be explained by a publication bias. The present review holds, however, also some limitations. First, the search for relevant articles was conducted in only two databases, albeit the most important ones in the field of psychological and educational research. Second, owing to the timing of the actual search, only articles published up to November 2019 were analyzed. Third, intervention studies were not included because they felt out of the scope of our research. However, to complete the picture presented, these limitations were defeated by adding relevant papers here the current discussion section that were published recently, not indexed in the databases we scanned, and discussing interventions.

The majority of the studies revealed by our systematic review (*n* = 22/37) used comprehensive tests to measure children's mathematical skills, compared with only 11 studies that used specific tasks. Four included both types of mathematical measures. Among the 22 studies that tested children with comprehensive batteries, 15 of them used questionnaires, five used observations, and two included both measures to assess home numeracy. Studies have shown that home learning environment (Melhuish et al., 2008; Niklas and Schneider, 2017; Visser et al., 2019; *n* = 4, Baker, 2015), informal (*n* = 1, Niklas and Schneider, 2014), and formal home numeracy (Huntsinger et al., 1997, 2016; Kleemans et al., 2012; Segers et al., 2015; Napoli and Purpura, 2018; *n* = 6, Anders et al., 2012) were positively associated with composite mathematical scores. Furthermore, two studies distinguished between basic and advanced formal activities and showed that advanced but not basic activities were related with children's composite mathematical scores (Del Rio et al., 2017; Zippert and Ramani, 2017). In line with the findings from the questionnaires, studies based on observational methods also demonstrated that home numeracy talk (*n* = 3, Susperreguy and Davis-Kean, 2016; Lehrl et al., 2019; Leyva, 2019), especially the advanced talk but not the basic talk (*n* = 2, Ramani et al., 2015; Elliott et al., 2017), was positively associated with children's composite math score.

Out of the 11 studies that tested children with specific mathematical measures, seven used questionnaires, three used observations, and only one used both measures to index home numeracy. The home learning environment (*n* = 1, Dearing et al., 2012), informal (*n* = 4, Benavides-Varela et al., 2016; Huang et al., 2017; Mutaf-Yildiz et al., 2018b; Vasilyeva et al., 2018), and formal home numeracy (*n* = 5, Kleemans et al., 2013, 2018; Huang et al., 2017; Mutaf-Yildiz et al., 2018a; Vasilyeva et al., 2018) were positively associated with specific mathematical tasks (i.e., calculations and counting). Moreover, studies using observations showed that home numeracy talk (*n* = 2, Levine et al., 2010; Glenn et al., 2018), especially the advanced talk (*n* = 1, Gunderson and Levine, 2011), was related with the specific mathematical skills, i.e., cardinal-principle knowledge.

All of the four studies that examined children with both comprehensive mathematical tests and specific mathematical tasks used questionnaires to assess home numeracy. Their results showed that informal (LeFevre et al., 2009) and advanced formal activities (Skwarchuk et al., 2014) were associated with composite mathematical scores. Moreover, both formal (LeFevre et al., 2009; Manolitsis et al., 2013) and informal activities (LeFevre et al., 2009; Skwarchuk et al., 2014) were related with some specific mathematical measures (i.e., math fluency, counting, and non-symbolic calculation).

Overall, only four studies (Zhou et al., 2006; Missall et al., 2015; Cheung et al., 2018; Leyva et al., 2019) did not observe any relationship between home numeracy (both questionnaire and observations) and mathematical skills (both comprehensive and specific mathematical measures). In addition, one study showed that the relationship between formal home numeracy and composite mathematical score was negative (Ciping et al., 2015). Although there are some commonalities, such as age, socio-economic status, and country in which the study was conducted, among these studies, these factors are probably not conclusive enough to explain the absence and negative direction of the relationship between home numeracy and mathematical skills. With respect to age, at first sight, in the five studies mentioned earlier, children were, on average, either slightly younger or older compared with the children in other studies (~5.5 years on average) that did observe a positive relation. In Cheung et al. (2018), Missall et al. (2015), and Zhou et al. (2006), children's mean age was 4.3, 4.5, and 4 years, respectively. The children in Leyva et al. (2019) were 6.6 years on average, which is older than most of the other studies. The age factor applies to the study of Ciping et al. (2015) that found a negative relationship and assessed home numeracy when children were on average 7.7 years old. This is later than all the other studies that collected home numeracy data when children were attending kindergarten. This seems to suggest there is a kind of critical period for home numeracy activities to result in a positive effect. However, this picture is not confirmed by all studies: two other studies (Ramani et al., 2015; Zippert and Ramani, 2017) that examined children's math skills and home numeracy at the age of around 4 and a study by Dearing et al. (2012), examining children's math skills and home numeracy at the age of around 6.7 reported positive associations (see also, Cheung et al., 2020). The number of studies examining relatively younger and older children is too small to establish reliable conclusions about the role of children's age.

Other commonalities between the studies in which a negative or no relation was found between home numeracy and children's mathematical skills are the socio-economic status of the family and the country where the research was conducted. The SES of the samples in those studies was low (Cheung et al., 2018; Leyva et al., 2019) or at least very diverse, with approximately half of the families having a low-SES background (Zhou et al., 2006; Ciping et al., 2015; Missall et al., 2015). Moreover, three (Zhou et al., 2006; Ciping et al., 2015; Cheung et al., 2018) of these five studies were conducted in Asian countries, i.e., Philippines and China, and one study included a sample living in the USA and having diverse ethnic backgrounds, 28% of which were Chinese (Leyva et al., 2019). However, the moderating effect of SES/country on the relation between home numeracy and mathematical skills is, just as age, not consistent. Huang et al. (2017) reported a positive relationship between home numeracy and children's mathematical skills in a Chinese sample [Huang et al., 2017; see also Zhang et al. (2020) for a recent replication]. Moreover, three studies with families from low socio-economic backgrounds (Ramani et al., 2015; Niklas and Schneider, 2017; Leyva, 2019; see also Harris et al., 2014) showed a positive association between home numeracy and children's mathematical skills.

Although the pattern revealed by our systematic review with respect to the moderating effect of SES/country on the relation between home numeracy and mathematical skills is not consistent and needs to be investigated further, it is clear that these factors are related to the frequency of home numeracy activities and children's mathematical skills. The majority of studies found a positive relation between SES and home numeracy activities (except Niklas and Schneider, 2014; Del Rio et al., 2017). In two studies by LeFevre et al. that were not included in our systematic analysis because they were not mentioned in the two databases we examined, it was shown that cultural factors indeed play a role in the relationship between home numeracy and mathematical skills. In one study, LeFevre et al. (2010b) found that Greek parents reported numeracy activities less frequently than Canadian parents, although formal home numeracy activities were associated with children's mathematical skills in both samples. In the other study, LeFevre et al. (2002) showed that French-speaking Canadian parents reported less frequent home numeracy activities than English-speaking Canadians.

In sum, the interactions between age, culture, home numeracy, and mathematical skills is worthy of further investigation, and may help to disentangle the precise moderating effects of age, SES, and culture on the relation between home numeracy and children's mathematical skills.

Beyond the fact that most of the studies have reported a positive relationship between home numeracy and children's mathematical skills, a more qualitative screening revealed five common findings and additional avenues for further research. First, six studies distinguished between basic and advanced home numeracy (either activities or talk) and all these studies have showed that advanced but not basic home numeracy plays an important role in children's mathematical skills (Gunderson and Levine, 2011; Skwarchuk et al., 2014; Ramani et al., 2015; Del Rio et al., 2017; Elliott et al., 2017; Zippert and Ramani, 2017). In these studies, examples of basic home numeracy included counting, reciting numbers, and identifying numerals, especially smaller than four. In combination with the fact that the children's age in these studies ranged from 4 to 6 (see Table 1), it can be assumed that these children already possessed this basic number knowledge. Based on this assumption, our results are in line with Vygotsky's theory of Zone of Proximal Development (ZPD, 1978): Practicing skills that children can already do by themselves does not result in improvement; by contrast, practicing skills that are just above the expertise level of the children provides opportunities for improvement. Following this argument, it can be hypothesized that basic home numeracy activities are more related to mathematical skills than advanced activities in relatively younger samples, whereas the opposite pattern is to be expected in relatively older samples. This needs to be confirmed in future research.

The second common point we observed is that six studies acquired home numeracy data from mothers, whereas only two of them also recruited fathers. The other studies did not explicitly distinguish between mothers and fathers, and gathered data from any parent whoever is the respondent which were mostly mothers. Five studies with mothers found associations between home numeracy and children's mathematical skills (Baker, 2015; Susperreguy and Davis-Kean, 2016; Del Rio et al., 2017; Elliott et al., 2017; Huang et al., 2017), and only one did not observe a relationship (Leyva et al., 2019). However, the two studies (Del Rio et al., 2017; Huang et al., 2017) that compared mothers and fathers showed that only mothers' but not fathers' formal home numeracy activities were linked with the children's mathematical skills. Del Rio et al. (2017) also reported that mothers and fathers did not significantly differ in the frequency of engaging in *formal* numeracy activities, whereas Huang et al. (2017) observed that mothers' engagement in formal numeracy activities was significantly more frequent than fathers' engagement. Furthermore, Huang et al. (2017) showed that fathers' engagement in *informal* activities was significantly more frequent than mothers' and that it was related with children's written calculation skills. To date, there are only two studies that compared mothers' and fathers' home numeracy explicitly and more research comparing mothers' and father's home numeracy activities is needed before drawing conclusions. However, it is an interesting topic worthwhile to explore further in future studies.

As the third common finding, we noticed that among the studies that used questionnaires, the number of investigations on informal home numeracy activities (*n* = 11) was smaller compared with investigations on formal home numeracy activities (*n* = 22). Some studies that investigated both formal and informal home numeracy in one sample showed that children's mathematical skills were related with formal home numeracy activities but not with informal ones (LeFevre et al., 2010b; Rosales et al., 2020), whereas others observed the reverse pattern: informal but not formal activities were associated with children's mathematical skills (Zhang et al., 2020). Still other studies reported that formal and informal activities were associated with different mathematical measures (e.g., LeFevre et al., 2009; Skwarchuk et al., 2014; Ciping et al., 2015; Mutaf-Yildiz et al., 2018b), or that the relation depends on the parent who completed the questionnaire (e.g., Del Rio et al., 2017). Moreover, studies that calculated home numeracy as the sum score of both formal and informal activities (Missall et al., 2015) found no relation between home numeracy and children's mathematical skills (see also Blevins-Knabe and Musun-Miller, 1996). Two studies reported that formal home numeracy activities were related with a wider range of mathematical skills in children compared with informal ones (Ramani et al., 2015; Huntsinger et al., 2016). However, a recent meta-analysis of 11 studies showed that informal activities are stronger predictors of mathematical skills compared with formal ones (Dunst et al., 2017). On the other hand, intervention studies (which were not analyzed in the current review) also revealed some conflicting results. Some studies showed playing number games, i.e., informal activities, improved children in mathematical skills (e.g., Ramani and Siegler, 2008; Siegler and Ramani, 2008); whereas others did not observe this effect (Vandermaas-Peeler et al., 2012; Zippert et al., 2019). Overall, both types of home numeracy activities seem to play a role in children's mathematical skills as supported by the recent findings of Susperreguy et al. (2020). Future research is encouraged to include both formal and informal home numeracy activities to better understand the reasons of the differential relations between formal and informal activities and children's mathematical skills.

Fourth, we noticed that observations of parent–child interactions (*n* = 8) were less frequently used to index home numeracy compared with questionnaires (*n* = 26). The more frequent use of questionnaires is understandable. It is less time consuming than observations, thus easily applicable to larger samples. However, responses on questionnaires are retrospective and rely on memory, thus might be influenced by social desirability bias or false memories (e.g., Gravetter and Forzano, 2006). On the other hand, observation studies are less influenced by memory and social desirability bias if a cover story is presented to hide the aim of the study (Harmon-Jones et al., 2007). These technical differences between the two methods call for more research directly comparing them to understand whether they can be used interchangeably or whether they measure different aspects of home numeracy. To date, only four studies used two methods of measuring home numeracy (questionnaire and observations) in one sample. Two of these studies showed that data from a questionnaire on the one hand and from observations on the other were not related (Missall et al., 2017; Mutaf-Yildiz et al., 2018b). In addition, Mutaf-Yildiz et al. (2018b) showed that children's mathematical skills were positively correlated with reported home numeracy activities, whereas it was negatively correlated with observed numeracy talk (see also, Zippert et al., 2019). Also, the other studies observed differences between both methods: Lehrl et al. (2019) found that children's mathematical skills were related with observed numeracy talk but not with formal numeracy activities. Ramani et al. (2015) found that both measures were differentially associated with various mathematical skills: Home numeracy measured with a questionnaire was related with a composite score of basic mathematics, whereas home numeracy measured with observations was related with a composite score of advanced mathematical skills. More research comparing the two methods is required to identify the underlying reasons of their unrelated outcomes and their differential relations to various math measures. It is especially important to reveal what aspects of home numeracy are exactly being measured via the two methods. For instance, it could be tested whether parents who indicate that they play board games with their children frequently on a questionnaire also have frequent number talk observed during a board game session with their children.

Finally, the fifth common finding we noticed is that most of the studies examined children with comprehensive mathematical tests (*n* = 22) compared with specific mathematical tasks (*n* = 11)—excluding the ones that used both methods (*n* = 4). However, the use of composite mathematical scores in home numeracy research makes it unclear to observe which specific skills are associated with home numeracy. For instance, Manolitsis et al. (2013) found that formal home numeracy activities were not associated with composite math score (TEMA-3); however, they were related to counting skills in kindergartners (see also Cheung et al., 2018). Furthermore, recent studies showed that it is important to dissociate between different mathematical skills as they found that formal and informal home numeracy activities were differentially related to specific mathematical tasks (Mutaf-Yildiz et al., 2018a; Vasilyeva et al., 2018).

There are two possible reasons for the differential associations. On the one hand, various numerical skills might rely on (partially) different underlying processes (e.g., Reynvoet and Sasanguie, 2016; Sasanguie et al., 2017). For example, when comparing effect sizes, the relation between mathematics and symbolic number processing is larger and more consistent compared with the effect size of the relation between mathematics and non-symbolic number processing (De Smedt et al., 2013; Schneider et al., 2017). On the other hand, different tasks assumed to measure one concept are not necessarily associated. It has for instance been shown that non-symbolic comparison and number line estimation tasks were not related to each other (Sasanguie and Reynvoet, 2013; Maertens et al., 2016). From these insights, it follows that home numeracy can be correlated with one specific skill but not another. Therefore, future research is needed to clarify which specific type of home numeracy activity is linked with which specific type of mathematical skills, by examining various specific types of mathematical skills.

The present systematic review comes with some limitations due to the decisions made in the search process. First, interest in home numeracy is increasing very fast and new papers are published frequently. The scope of the articles analyzed in this research is limited to the ones published until November 2019. Second, several studies—including some that we were aware of—have been missed in our search because we screened only two databases and they were not indexed in those databases. To stick to the search strategy, those articles were not included in the analysis. Third, the scope of our research excluded home numeracy intervention studies. In intervention studies, parents are informed about the role they play in the development of their children's mathematical skills and how they can improve their support. Results suggest that home numeracy interventions have a positive effect on mathematical skills (e.g., Starkey and Klein, 2000; Niklas et al., 2016). However, in order to present a complete picture, the discussion was expanded with those recently published, missed, and discussing interventions papers (LeFevre et al., 2002, 2010a,b; Vandermaas-Peeler et al., 2012; Harris et al., 2014; Dunst et al., 2017; Cheung et al., 2020; Susperreguy et al., 2020; Zhang et al., 2020; Zippert et al., 2020). Finally, it should be noted that the current results did not inform us about the overall effect size of the relationship between home numeracy and mathematical skills in children.

In conclusion, the current systematic search and review demonstrated that a positively significant link between home numeracy and children's mathematical skills has been observed in the majority of the studies. In addition, a *p*-curve analysis confirmed that this relationship holds a true effect. Moreover, a qualitative inspection of all studies revealed some possible sources for the variance in the relationship between home numeracy and mathematical skills in children across studies. These sources include children's age, family SES, and location of the research, the distinction between basic and advanced activities, differences between mothers and fathers, differences between questionnaires and/or observations, and differences between formal and informal activities. Differences between studies also emerged due to differences in the measurement of mathematical skills, i.e., specific mathematical tasks or comprehensive mathematical tests. These sources all seem to have an impact on observed findings. Therefore, more research is necessary to draw quantitative conclusions about these possible sources of variance.

## Data Availability Statement

All datasets presented in this study are included in the article and further inquiries can be directed to the corresponding author.

## Author Contributions

BMY, DS, BDS, and BR conceived and designed the study and interpreted the results. BMY organized the data and ran the analyses, wrote the draft of the overall study, and revised the draft carefully. DS, BDS, and BR critically reviewed the draft. All authors contributed to the article and approved the submitted version.

## Conflict of Interest

The authors declare that the research was conducted in the absence of any commercial or financial relationships that could be construed as a potential conflict of interest.
